# Editorial: Reviews in healthcare professions education

**DOI:** 10.3389/fmed.2024.1362030

**Published:** 2024-05-14

**Authors:** Ahsan Sethi, Madawa Chandratilake

**Affiliations:** ^1^Health Professions Education, QU Health, Qatar University, Doha, Qatar; ^2^Faculty of Medicine, University of Kelaniya, Ragama, Sri Lanka

**Keywords:** arthroscopy, BOPPPS, communication skill, health profession education, reviews

Healthcare is a complex and ever-evolving field, encompassing various challenges, advancements, and intricacies that impact patient care, medical education, and clinical practice. Review articles play a crucial role in shedding light on different aspects of healthcare, offering valuable insights into emerging trends, innovative methodologies, and potential areas for improvement. In this editorial, we delve into five review articles that explore diverse facets of healthcare, ranging from evidence translation and patient-physician relationships to clinical reasoning and training methodologies. Through an analysis of these articles, we aim to gain a deeper understanding of the multifaceted nature of healthcare and the implications for medical education and practice.

The review articles encompass a wide range of topics, each offering unique perspectives on key aspects of healthcare. One article (Lyu and Li) explores the challenges of translating evidence into practice and the role of clinical practice guidelines in guiding healthcare decisions. It emphasizes the importance of evidence-based practices in improving patient outcomes while highlighting the barriers and limitations in implementing these practices effectively. Another article (Keller et al.) focuses on the evolving patient-physician relationship, particularly in the context of technological advancements. It examines how technology influences communication between patients and healthcare providers, shaping the dynamics of care delivery and patient engagement. The article underscores the need for maintaining personalized, patient-centered care amidst the increasing reliance on digital health tools and telemedicine. Clinical reasoning, a fundamental aspect of healthcare practice, is explored in depth in another article (Vreugdenhil et al.). The review delves into the complexities of clinical reasoning processes among healthcare professionals, highlighting the differences between physicians and nurses in their approaches to problem-solving and decision-making. It emphasizes the importance of understanding these differences to foster effective collaboration and optimize patient care outcomes. Wrist arthroscopy, a specialized surgical technique for diagnosing and treating wrist injuries, is the focus of another review article by Shi et al.. The review examines the advantages and challenges associated with wrist arthroscopy and explores evolving methodologies in training programs for surgeons. It highlights the importance of anatomical knowledge, operative skills, and innovative training methods in ensuring optimal surgical outcomes and patient safety. The review article by Ma et al. investigates the application of the BOPPPS teaching strategy in medical education in China. It focuses on determining the effectiveness of the BOPPPS strategy in enhancing student engagement and learning outcomes compared to traditional lecture-based methods. This review underscores the potential of innovative teaching approaches in improving medical education delivery and preparing future healthcare professionals for clinical practice.

Methodologically, each review article adopts a distinct methodological approach tailored to its research objectives and focus areas. For example, the article by Lyu and Li employs a systematic search methodology, spanning a 10-year period and incorporating gray literature to ensure comprehensive coverage of relevant studies. In contrast, Keller et al. utilize a narrative exploration approach, weaving together patient stories and clinical anecdotes to illustrate the nuances of the patient-physician relationship. Vreugdenhil et al. employ a structured analytical framework, the “onion model,” to dissect clinical reasoning processes into layers, offering a comprehensive understanding of this complex phenomenon. The fourth article synthesizes existing research on wrist arthroscopy training, examining a wide range of methodologies and training programs to identify best practices in surgical education. Lastly, Ma et al. utilize a meta-analysis and systematic review approach, adhering to established guidelines such as PRISMA and PICOS to evaluate the effectiveness of the BOPPPS teaching strategy. It conducts a rigorous analysis of available evidence, including the risk of bias assessment and statistical analysis, to draw meaningful conclusions regarding the impact of innovative teaching methods on medical education outcomes.

Collectively, these review articles provide valuable insights into the multifaceted nature of healthcare and its implications for medical education and practice. They underscore the importance of evidence-based practices, personalized patient care, interdisciplinary collaboration, and innovative teaching methodologies in addressing the evolving challenges and demands of healthcare. Moving forward, further research is needed to explore the effectiveness of different educational strategies, the impact of technology on patient-provider relationships, and the optimization of clinical reasoning processes across healthcare professions. Additionally, ongoing evaluation and refinement of training methodologies, such as wrist arthroscopy training programs, are essential to ensure the continued advancement of surgical education and patient care.

In summary, the review articles offer an exploration of various aspects of healthcare, from evidence translation and patient-physician relationships to clinical reasoning and surgical training. By examining these diverse topics through different methodological lenses, we gain valuable insights into the complexities and challenges of healthcare delivery and medical education. Moving forward, continued research, and innovation are essential to address the evolving needs of patients, healthcare professionals, and healthcare systems worldwide.

## Author contributions

AS: Supervision, Conceptualization, Writing – original draft, Writing – review & editing. MC: Writing – original draft, Writing – review & editing.

